# Prognostic value of coagulation tests for in-hospital mortality in patients with traumatic brain injury

**DOI:** 10.1186/s13049-017-0471-0

**Published:** 2018-01-05

**Authors:** Qiang Yuan, Jian Yu, Xing Wu, Yi-rui Sun, Zhi-qi Li, Zhuo-ying Du, Xue-hai Wu, Jin Hu

**Affiliations:** Department of Neurosurgery, Huashan Hospital, Fudan University, 12 Wulumuqi Zhong Road, Shanghai, 200040 People’s Republic of China

**Keywords:** Traumatic brain injury, Coagulopathy, Coagulation tests, Mortality, Prediction model

## Abstract

**Background:**

Coagulopathy is commonly observed after traumatic brain injury (TBI). However, it is not known whether using the standard independent predictors in conjunction with coagulation tests would improve their prognostic value. We determined the incidence of TBI-associated coagulopathy in patients with isolated TBI (iTBI), evaluated the prognostic value of coagulation tests for in-hospital mortality, and tested their predictive power for in-hospital mortality in patients with iTBI.

**Methods:**

We conducted a retrospective, observational database study on 2319 consecutive patients with iTBI who attended the Huashan Hospital Department of the Neurosurgery Neurotrauma Center at Fudan University in China between December 2004 and June 2015. Two models based on the admission characteristics were developed: model A included predictors such as age, Glasgow Coma Scale (GCS) score, pupil reactivity, type of injury, and hemoglobin and glucose levels, while model B included the predictors from model A as well as coagulation test results. A total of 1643 patients enrolled between December 2004 and December 2011 were used to derive the prognostic models, and 676 patients enrolled between January 2012 and June 2015 were used to validate the models.

**Results:**

Overall, 18.6% (*n* = 432) of the patients developed coagulopathy after iTBI. The prevalence of acute traumatic coagulopathy is associated with the severity of brain injury. The percentage of platelet count <100 × 109/L, international normalized ratio (INR) > 1.25, the prothrombin time (PT) > 14 s, activated partial thromboplastin time (APTT) > 36 s, D-dimer >5 mg/L and fibrinogen (FIB) < 1.5 g/L was also closely related to the severity of brain injury, significance being found among three groups. Age, pupillary reactivity, GCS score, epidural hematoma (EDH), and glucose levels were independent prognostic factors for in-hospital mortality in model A, whereas age, pupillary reactivity, GCS score, EDH, glucose levels, INR >1.25, and APTT >36 s exhibited strong prognostic effects in model B. Discrimination and calibration were good for the development group in both prediction models. However, the external validation test showed that calibration was better in model B than in model A for patients from the validation population (Hosmer–Lemeshow test, *p* = 0.152 vs. *p* = 0.046, respectively).

**Conclusions:**

Coagulation tests can improve the predictive power of the standard model for in-hospital mortality after TBI.

## Background

Traumatic brain injury (TBI) is the leading cause of morbidity and disability in trauma patients and is responsible for a significant proportion of traumatic deaths in young adults [1, 2]. Coagulopathy is commonly observed after TBI [3–5]. Although the incidence of coagulopathy is strongly associated with the severity of the injury, coagulopathy itself exerts an independent effect on mortality [4, 6, 7].

The reported incidence of TBI-associated coagulopathy ranges from 10 to 87.5% [8–10]. The wide range of values reflects the lack of a standard definition for coagulopathy. Other factors contributing to the variability in incidence rates include differences in the patient populations evaluated, blood sampled at different time points, and the use of various coagulation assays. Moreover, studies investigating the same coagulation marker may use different cutoff values or sensitivity levels, thereby limiting generalizability. A recent meta-analysis of 22 studies found the overall incidence of TBI-associated coagulopathy to be 35.2% [11]. A previous study found that the presence of coagulopathy was associated with a nine-fold increase in the odds for mortality and increased the likelihood of a poor outcome by a factor of 36 [9]. Thus, it is clear that the development of coagulopathy after TBI is significantly associated with increased mortality and poor outcomes [12, 13].

Standard laboratory tests used to measure hemostasis and bleeding risk in patients with TBI include the international normalized ratio (INR), prothrombin time (PT), activated partial thromboplastin time (APTT), and platelet counts (PLT). D-dimer and fibrinogen (FIB) levels may provide additional useful data; however, their use is not routine. PT and APTT were originally developed to measure the in vitro activity of specific coagulation factors; however, they are currently used to predict the bleeding risk in perioperative neurosurgical patients [14]. The coagulation panel and PLT may also be used to predict the bleeding risk.

Some admission predictors such as age, absence of pupillary reactivity, the Glasgow Coma Scale (GCS) score, and CT characteristics have been routinely used to predict outcome in patients with TBI [15]. Although coagulation abnormalities may be a better predictor of mortality than the absence of the bilateral pupillary light reflex in some patients [16], prognosis is rarely predicted by coagulation status alone in the clinical setting. However, it is not known whether using the standard independent predictors in conjunction with coagulation tests would improve their prognostic value.

The aims of our study were two-fold: first, to determine the incidence of TBI-associated coagulopathy in patients with isolated TBI (iTBI) who attended an adult neurotrauma center; second, to evaluate the prognostic value of coagulation tests with respect to in-hospital mortality and to test their predictive power in prediction models for in-hospital mortality in patients with iTBI. Furthermore, we performed validation tests to assess the internal and external validity.

## Methods

### Patient population

Two thousand three hundred nineteen consecutive patients with iTBI who attended the Huashan Hospital Department of the Neurosurgery Neurotrauma Center at Fudan University in China between December 2004 and June 2015 were retrospectively collected in this study. The inclusion criteria were as follows: TBI with radiological signs of intracranial brain injury (epidural or subdural hematoma [EDH or SDH], intraparenchymal hemorrhage [IPH], contusion, or subarachnoid hemorrhage [SAH]) documented using computed tomography (CT); ≥14 years of age; and admission within 24 h of TBI. Patients with traumatic injury to a body region other than the brain with an Abbreviated Injury Severity score ≥ 3, a penetrating brain injury, preexisting coagulapthy or concurrent use of anticoagulant or antiplatelet agents were excluded from the study. All patients were evaluated and treated according to the Guidelines for the Management of Severe Head Injury. The course of the study was authorized from the Ethical Committee of our institution.

### Demographic data and coagulation tests

Clinical and demographic characteristics, including age, sex, mechanism of injury, pupillary reaction to light, GCS score at admission, use of an intracranial pressure monitor, decompressive craniectomy (DC), and length of stay (LOS) were recorded for all patients. Moreover, the results of the initial CT scan on admission were used to assess the severity and type of injury.

PLT and coagulation tests, including INR, PT, APTT, and FIB and D-dimer levels, were performed in all patients within 12 h of injury and assessed at the Huashan Hospital Central Clinical Chemistry Laboratory using routine laboratory assays. We carefully examined the distributions of the coagulation tests, and the shape of the relationships between the continuous variables and mortality were examined by univariate analysis with a non-linear correlation (cubic spline functions). These relationships were continuous with no clear indication of threshold values. To obtain comparable odds ratios for the relationships, we rescaled each variable as follows: PT ≤14 to >14 s, APTT ≤36 to >36 s, INR ≤1.25 to >1.25, D-dimer level < 1, 1–5 to >5 mg/L, and FIB ≤1.5 to >1.5 g/L. PLT was classified as normal (≥100 × 10^9^/L) and low (<100 × 10^9^/L). Coagulopathy was defined as one or more of the following: PLT <100 × 10^9^/L, INR >1.25, PT >14 s, and APTT >36 s. Furthermore, hemoglobin (Hb), hematocrit (HCT) and glucose levels were measured and recorded. The main outcome measure was in-hospital mortality.

### Statistical analysis

Continuous variables were expressed as means ± standard deviation or medians (interquartile range) and categorical variables as percentages. The univariate analyses of categorical data were performed using the chi-squared test. Equality of variance was assessed using Levene’s test. Normally distributed variables were compared using Student’s *t*-tests or analysis of variance, whereas non-normally distributed variables were compared using the Kruskal-Wallis or Mann–Whitney *U*-tests. A univariate analysis with non-linear correlation (cubic spline functions) was used to evaluate the shape of the relationship between the continuous variables and outcome.

The prognostic models were derived from the data of 1643 patients recruited between December 2004 and December 2011. Following the univariate analyses, a forward stepwise logistic regression analysis of in-hospital mortality was used to develop the prediction models. Two models for in-hospital mortality were developed based on admission characteristics: model A included standard predictors such as age, GCS score, pupil reactivity, type of injury, Hb, and glucose levels, and model B included the results of the coagulation tests in addition to the predictors from model A.

Performance of the models was assessed according to discrimination, by means of the *c* statistic (equivalent to the area under the receiver operator characteristic curve) and calibration, using the Hosmer–Lemeshow (H-L) goodness-of-fit test. The bootstrap resampling method was used to assess the internal validity of our models [17]. External validation were assessed using an external series of 676 patients with iTBI who were recruited between January 2012 and June 2015. The *c* statistic was used to assess discrimination and a smooth, nonparametric calibration line created using the LOWESS algorithm was used to assess calibration graphically in the models. The H-L test used the R code function written by Steyerberg [17]. The R statistical package for Windows version 2.12.1 (The R Foundation for Statistical Computing) was used to conduct the statistical tests. *P*-values <0.05 were deemed to indicate statistical significance.

## Results

Overall, 18.6% (*n* = 432) of the patients in our study developed coagulopathy after iTBI. Coagulopathy developed in 30.4% of patients with severe iTBI and in 11.4% (*n* = 126) of patients with mild iTBI. The prevalence of acute traumatic coagulopathy is associated with the severity of the brain injury. We observed an INR >1.25 in 5.8% of patients, PT >14 s in 8.1%, APTT >36 s in 5.6%, PLT <100 × 10^9^/L in 10.7%, FIB level < 1.5 g/L in 15.3%, and D-dimer level > 5 mg/L in 22.1% of patients. These percentages were closely associated with the severity of brain injury, with significance detected among the three groups. Patients with severe TBI had a significantly higher median INR, PT, APTT, D-dimer level and lower PLT and FIB level than those with milder injuries (Table 1).

**Table 1:** Summary of patient characteristics and coagulation tests by the severity of TBI

	Severe injury (GCS 3–8)	Moderate injury (GCS 9–12)	Mild injury (GCS 13–15)	Total
N	662	547	1110	2319
Age (yrs) (mean ± SD)	47.84 ± 16.03	48.07 ± 15.95	47.05 ± 17.03	47.52 ± 16.50
Sex				
Male	513 (77.5)	430 (78.6)	819 (73.8)	1762 (76.0)
Female	149 (22.5)	117 (21.4)	291 (26.2)	557 (24.0)
Mechanism of injury				
Motor vehicle accident	422 (63.7)	334 (61.1)	593 (53.4)	1349 (58.2)
Fall	99 (15.0)	78 (14.3)	147 (13.2)	324 (14.0)
Stumble	86 (13.0)	83 (15.2)	219 (19.7)	388 (16.7)
Blow to head	32 (4.8)	29 (5.3)	113 (10.2)	174 (7.5)
Others	23 (3.5)	23 (4.2)	38 (3.4)	84 (3.6)
Pupillary reactions*				
Both reacting	387 (58.5)	528 (96.5)	1110 (100)	2025 (87.3)
One reacting	195 (29.5)	19 (3.5)	0 (0)	214 (9.2)
None reacting	80 (12.1)	0 (0)	0 (0)	80 (3.4)
Type of injury				
SDH*	263 (39.7)	154 (28.2)	231 (20.8)	648 (27.9)
EDH	184 (27.8)	152 (27.8)	304 (27.4)	640 (27.6)
IPH*	526 (79.5)	435 (79.5)	610 (55.0)	1571 (67.7)
tSAH*	405 (61.2)	311 (56.9)	521 (46.9)	1237 (53.3)
DAI*	60 (9.1)	11 (2.0)	3 (0.3)	74 (3.2)
Skull fracture*	88 (13.3)	110 (20.1)	265 (23.9)	463 (20.0)
INR*	1.08 (1.02-1.16)	1.05 (1.00–1.12)	1.03 (0.99–1.08)	1.05 (1.00–1.12)
INR > 1.25*	76 (11.5)	27 (4.9)	31 (2.8)	134 (5.8)
PT(s)*	12.4 (11.8-13.4)	12.0 (11.4–12.8)	11.8 (11.2–12.3)	12.0 (11.3–12.8)
PT > 14 s*	100 (15.1)	36 (6.6)	51 (4.6)	187 (8.1)
APTT(s)*	26.1 (23.5–29.8)	25.0 (22.0–28.8)	24.7 (22.0–27.5)	25.0 (22.4–28.5)
APTT > 36 s*	64 (9.7)	24 (4.4)	43 (3.9)	131 (5.6)
FIB(g/L)*	2.1 (1.5–3.1)	2.3 (1.8–3.1)	2.5 (1.9–3.1)	2.3 (1.8–3.1)
FIB < 1.5 g/L*	174 (26.3)	82 (15.0)	98 (8.8)	354 (15.3)
D-dimer (mg/L)*	2.856 (0.840–7.080)	2.101 (0.852–5.174)	0.879 (0.300–2.451)	1.552 (0.453–4.298)
D-dimer <1 mg/L*	186 (28.1)	154 (28.2)	591 (53.2)	931 (40.1)
D-dimer 1–5 mg/L	238 (36.0)	249 (45.5)	388 (35.0)	875 (37.7)
D-dimer >5 mg/L	238 (36.0)	144 (26.3)	131 (11.8)	513 (22.1)
PLT(×10^9^/L)*	158 (115–204)	167 (129-210)	178 (147–213)	171 (134–210)
PLT < 100 × 10^9^/L*	115 (17.4)	63 (11.5)	69 (6.2)	247 (10.7)
Coagulopathy*	201 (30.4)	105 (19.2)	126 (11.4)	432 (18.6)
Hb(g/L)*	125 (108–141)	134 (117-146)	135 (123–147)	133 (117–145)
HCT(%)*	37.1(32.2-40.9)	38.8 (34.5–42.1)	39.5 (36.3–42.7)	38.7 (34.6–42.1)
Glucose(mmol/L)*	8.6 (7.3–10.4)	7.7 (6.7–9.2)	6.8 (6.0–8.0)	7.5 (6.4–9.0)
ICP monitoring*	447 (67.5)	233 (42.6)	80 (7.2)	760 (32.8)
Craniectomy*	365 (55.1)	136 (24.9)	39 (3.5)	540 (23.3)
Mortality*	131 (19.8)	27 (4.9)	16 (1.4)	174 (7.5)
LOS*	18 (11-28)	15 (10-22)	9 (6–14)	13 (8–20)

The univariate analyses of categorical data were performed with a chi-square test. Normally distributed variables were compared using ANOVA, whereas nonnormally distributed variables were compared using the Kruskal-Wallis test

*P < 0.05

The patient characteristics and outcomes for the coagulopathy and non-coagulopathy groups are shown in Table 2. The proportions of patients with none pupillary reactivity, IPH, ICP monitoring and craniectomy were comparatively high in the coagulopathy group and low in non-coagulopathy group. The glucose and LOS were higher in the coagulopathy group, whereas the GCS at admission and Hb levels were lower in the coagulopathy group. The in-hospital mortality rate was significantly higher in the coagulopathy compared with the non-coagulopathy group.

**Table 2:** Patients Characteristics and Outcome of the Coagulopathy and Non-coagulopathy Patients

	Coagulopathy (*n* = 432) n (%)	Non-coagulopathy (*n* = 1887) n (%)	*P* value
N	432	1887	
Age (yrs) (mean ± SD)	47.53 ± 17.16	47.51 ± 16.34	0.984
Sex			
Male	332 (76.9)	1430 (75.8)	0.639
Female	100 (23.1)	457 (24.2)	
Mechanism of injury			
Motor vehicle accident	273 (63.2)	1076 (57.0)	0.087
Fall	62 (14.4)	262 (13.9)	
Stumble	58 (13.4)	330 (17.5)	
Blow to head	25 (5.8)	149 (7.9)	
Others	14 (3.2)	70 (3.7)	
Pupillary reactions			
Both reacting	337 (78.0)	1688 (89.5)	<0.001
One reacting	61 (14.1)	153 (8.1)	
None reacting	34 (7.9)	46 (2.4)	
Type of injury			
SDH	137 (31.7)	511 (27.1)	0.053
EDH	126 (29.2)	514 (27.2)	0.419
IPH	330 (76.4)	1241 (65.8)	<0.001
tSAH	235 (54.4)	1002 (53.1)	0.626
DAI	19 (4.4)	55 (2.9)	0.114
Skull fracture	85 (19.7)	378 (20.0)	0.867
Injury severity(GCS at admission)(mean ± SD)	9 (6–13)	13 (9–15)	<0.001
GCS 3–8	201 (46.5)	461 (24.4)	<0.001
GCS 9–12	105 (24.3)	442 (23.4)	
GCS 13–15	126 (29.2)	984 (52.1)	
Hb(g/L)	119 (101–137)	135 (121–147)	<0.001
Glucose(mmol/L)	8.0 (6.6–9.8)	7.4 (6.3–8.8)	<0.001
ICP monitoring	193 (44.7)	567 (30.0)	<0.001
Craniectomy	167 (38.7)	373 (19.8)	<0.001
Mortality	76 (17.6)	98 (5.2)	<0.001
LOS	15 (8–25)	12 (8–19)	<0.001

The patient characteristics and outcomes for the model-development and validation groups are shown in Table 3. We found several significant between-group differences: the validation patients were older than those in the development group (mean age, 48.07 vs. 47.84 years, respectively), and the proportions of patients with bilateral pupillary reactivity, IPH, SAH, and a fractured skull were comparatively low in the development group and high in the validation patients. The proportions of those with diffuse axonal injury, PT >14 s and PLT <100 × 10^9^/L were high in the development compared with the validation group. The median glucose, HCT, and D-dimer levels were higher in the development patients, whereas the median INR, PT, and Hb levels were higher in the validation patients. The in-hospital mortality rate was not significantly different between groups.

**Table 3:** Patients Characteristics and Outcome of the Development Patients and the Validation Patients

	Development Patients (*n* = 1643) n (%)	Validation Patients (*n* = 676) n (%)	*P* value
N	1643	676	
Age (yrs) (mean ± SD)	47.84 ± 16.03	48.07 ± 15.95	0.042
Sex			
Male	1253 (76.3)	509 (75.3)	0.620
Female	390 (23.7)	167 (24.7)	
Mechanism of injury			
Motor vehicle accident	970 (59.0)	379 (56.1)	0.233
Fall	215 (13.1)	109 (16.1)	
Stumble	268 (16.3)	120 (17.8)	
Blow to head	127 (7.7)	47 (7.0)	
Others	63 (3.8)	21 (3.1)	
Pupillary reactions			
Both reacting	1413 (86.0)	612 (90.5)	0.011
One reacting	169 (10.3)	45 (6.7)	
None reacting	61 (3.7)	19 (2.8)	
Type of injury			
SDH	443 (27.0)	205 (30.3)	0.101
EDH	468 (28.5)	172 (25.4)	0.137
IPH	1076 (65.5)	495 (73.2)	<0.001
tSAH	811 (49.4)	426 (63.0)	<0.001
DAI	61 (3.7)	13 (1.9)	0.026
Skull fracture	280 (17.0)	183 (27.1)	<0.001
Injury severity			
GCS 3–8	486 (29.6)	176 (26.0)	0.180
GCS 9–12	388 (23.6)	159 (23.5)	
GCS 13–15	769 (46.8)	341 (50.4)	
INR	1.05 (1.00–1.11)	1.05 (1.00–1.12)	0.012
INR > 1.25	97 (5.9)	37 (5.5)	0.686
PT(s)	11.4 (10.9–12.1)	11.5 (10.9–12.3)	<0.001
PT > 14 s	152 (9.3)	35 (5.2)	0.001
APTT(s)	24.1 (21.4–26.7)	24.4 (21.9–27.9)	0.05
APTT > 36 s	98 (6.0)	33 (4.9)	0.305
FIB(g/L)	2.3 (1.7–3.2)	2.3 (1.7–3.1)	0.638
FIB < 1.5 g/L	247 (15.0)	107 (15.8)	0.629
D-dimer (mg/L)	5.005 (2.240–13.810)	3.230 (1.240–11.540)	<0.001
D-dimer <1 mg/L	729 (44.4)	202 (29.9)	<0.001
D-dimer 1–5 mg/L	641 (39.0)	234 (34.6)	
D-dimer >5 mg/L	273 (16.6)	240 (35.5)	
PLT(×10^9^/L)	177 (139–215)	171 (137–213)	0.999
PLT < 100 × 10^9^/L	189 (11.5)	58 (8.6)	0.038
Coagulopathy	331 (20.1)	101 (14.9)	0.003
Hb(g/L)	130 (112–144)	131 (115–144)	0.023
HCT(%)	38.6 (33.6–42.3)	38.5 (34.4–41.8)	0.003
Glucose(mmol/L)	7.4 (6.2–8.6)	7.2 (6.3–8.6)	0.001
ICP monitoring	509 (31.0)	251 (37.1)	0.004
Craniectomy	406 (24.7)	134 (19.8)	0.011
Mortality	128 (7.8)	46 (6.8)	0.413
LOS	11 (7–17)	11 (7–18)	<0.001

**Fig. 1:**
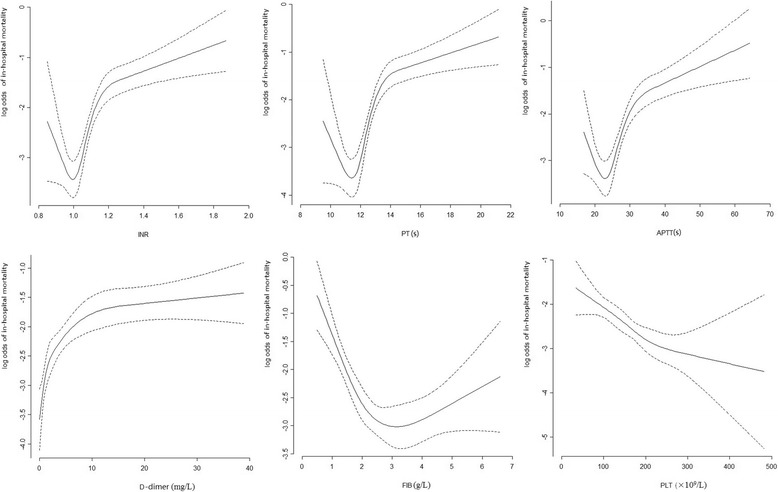
The shape of the relationship between continuous variables (coagulation tests) and in-hospital mortality. The solid line indicates that the relationship was analyzed with cubic spline function. The dash line indicates 95% CI

The univariate analysis revealed that all predictors were statistically significant with respect to in-hospital mortality. A nonlinear relationship was observed between PLT and the coagulation tests; thus, each variable was rescaled (Fig. 1).

The results of the multivariable logistic regression analysis are shown in Table 4. Age, pupillary reactivity, GCS score, EDH, and glucose levels were independent prognostic factors for in-hospital mortality in model A. In model B, age, pupillary reactivity, GCS score, EDH, glucose levels, INR >1.25, and APTT >36 s were strong prognostic indicators of in-hospital mortality. Epidural hemorrhage detected by CT was a relatively favorable sign, whereas INR >1.25 and APTT >36 s were associated with higher in-hospital mortality.

**Table 4:** Multivariable Logistic Regression Analysis of Association Between Predictors and in-hospital mortality

Predictors	Model A (Basic) (95% CI)	Model B (Basic + coagulation test) (95% CI)
Age	1.03 (1.02–1.05)	1.03 (1.02–1.05)
GCS	0.76 (0.71–0.82)	0.76 (0.70–0.82)
Pupillary reactions	1.93 (1.35–2.76)	1.67 (1.15–2.43)
EDH	0.38 (0.21–0.67)	0.37 (0.21–0.68)
Glucose	1.14 (1.08–1.21)	1.14 (1.07–1.20)
INR > 1.25	–	2.65 (1.34–5.23)
APTT > 36 s	–	3.25 (1.67–6.34)

We developed two prediction models for in-hospital mortality. The performance of each model is shown in Table 5. The discrimination for in-hospital mortality in the development group was good in both models (model A, *c* = 0.882 and model B, *c* = 0.893), and the H-L test revealed good calibration in both models (*p* > 0.05).

**Table 5:** Performance and Validation of Prediction Models

	In-hospital Mortality C Statistic (95%CI)	P^a^
Development(n = 1643)		
Model A	0.882 (0.855–0.909)	0.925
Model B	0.893 (0.865–0.920)	0.240
Internal Validation^b^		
Model A	0.878 (0.851–0.905)	–
Model B	0.890 (0.862–0.917)	–
External Validation(n = 676)		
Model A	0.868 (0.816–0.921)	0.046
Model B	0.875 (0.824–0.927)	0.152

^a^H-L tests

^b^Internal validation with 200 bootstrap re-samples using Harrell’s validation function

**Fig. 2:**
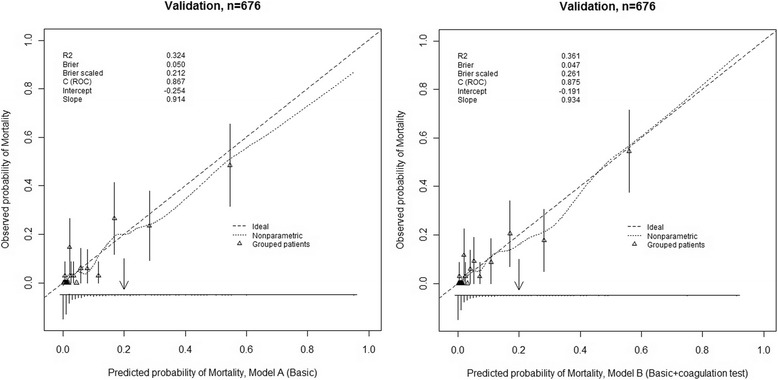
Validation of the prognostic models in validation patients (*n* = 676). The smooth solid curves reflect the relation between observed probability of in-hospital mortality and predicted probability of in-hospital mortality. The triangles indicate the observed frequencies by deciles of predicted probability

The internal validation test showed no over-optimism bias in the predictive *c* statistic of either model. The external validation test showed good discrimination for mortality in both predictive models (model A, *c* = 0.868 and model B, *c* = 0.875). However, calibration was better in model B than in model A (H-L test, *p* = 0.152 vs. *p* = 0.046, respectively). Thus, model B was generalizable and predicted in-hospital mortality in new patients more accurately compared with model A. Calibration curves for the outcomes are shown in Fig. 2.

## Discussion

We examined the prognostic value of admission coagulation tests with regard to in-hospital mortality after iTBI and developed a series of prognostic models to predict the probability of in-hospital mortality.

Multivariate logistic regression analysis revealed that age, pupillary reactivity, GCS, EDH, glucose levels, INR >1.25, and APTT >36 s were independently associated with in-hospital mortality. These variables can be readily obtained on admission to a neurosurgical unit and are consistent with prior studies of prognostic predictors [15]. Both of our prediction models, which were based on admission predictors, had excellent discrimination and calibration in the development group. Good generalizability is essential for predicting outcomes in new patients; thus, we assessed the external validity of our prognostic models to assess their generalizability. External validation confirmed that the prediction model using a combination of standard predictors and coagulation tests had better and more accurate calibration than that of the model based on standard predictors alone and had good generalizability. Thus, the most important and novel finding of our study is that the addition of coagulation test results to a multivariate logistic regression analysis can improve the predictive power of the standard prognostic model for in-hospital mortality. To the best of our knowledge, our study is the first to demonstrate the feasibility of this combined approach to predict outcomes in patients with TBI.

The ability to predict outcomes is crucial for effective care of patients with TBI [18, 19]. Information provided to relatives should be based on solid clinical and scientific evidence, which will help them prepare for the future and facilitate their understanding of the risky and potentially painful interventions that TBI patients may be required to undergo. Predictive systems promote quality assurance by providing a means for assessing patient care that can be used to make comparisons across or within institutions [20, 21]. The clinical value of predictors in an outcome prediction model is determined by their reliability on assessment, the prevalence of abnormalities, and the strength of the prognostic effect (odds ratios). The coagulation tests we investigated are standardized among laboratories and, thus, are objective and reliable. The prevalence of abnormal values was substantial for the coagulation tests investigated. The strongest predictive effects were observed for INR and APTT. Multiple associations were observed among coagulation tests and between coagulation tests and clinical parameters; however, the prognostic effects remained substantial following adjusted analysis, suggesting that the coagulation tests are of considerable prognostic relevance in TBI.

We found that 18.6% of the study population developed coagulopathy after iTBI, and 30.4% of the patients with severe iTBI experienced coagulopathy. These findings are consistent with previous reports [9, 10]. A meta-analysis of 22 studies found an overall incidence of TBI-associated coagulopathy of 35.2%; however, the definition of coagulopathy and the patient populations varied among the included studies [11].

Previous studies have shown that the most consistent coagulation abnormality is PT [7, 22]. PT reflects the activation time of the extrinsic, or tissue factor, pathway based on the cascade model of hemostasis. Most previous investigations of TBI-associated coagulopathy focused on PT or INR abnormalities [23, 24]. The International Mission on Prognosis and Analysis of Clinical Trials in TBI (IMPACT) study found that PT prolongation on admission was present in 221 of 850 patients (26%) and was associated with a 64% increase in mortality risk [12]. APTT reflects the activation time of the intrinsic, or contact activation, pathway and is particularly sensitive to deficiencies in coagulation factors IX, XI, and VIII. Although affected less often than the PT, APTT is more highly correlated with poor outcome and mortality than are other markers of coagulation [25, 26]. Thrombocytopenia on admission is a complication of TBI in fewer than 10% of cases [12, 27, 28]. In our study, 10.7% of patients had a PLT <100 × 10^9^/L. Thus, coagulation tests may provide more useful information on mortality after TBI than do the standard admission variables.

Recognition of the importance of coagulopathy in TBI is increasing. The mechanisms underlying TBI-associated coagulopathy are not well understood, although massive release of tissue factor, altered protein C homeostasis, microparticle upregulation, and platelet hyperactivity have been shown to play prominent roles [5, 29]. Hypocoagulable and hypercoagulable phenotypes have been identified in patients after TBI; however, their clinical significance, pathophysiological mechanisms, and temporal relationships are not well understood. Routine coagulation tests, such as PT, APTT, and PLT, demonstrate poor sensitivity to the disturbances associated with TBI-related coagulopathy and do not explain the observed hypercoagulability.

Although our results clearly indicate that coagulation tests may play a significant role in prognostic models and calculators for patients with TBI, caution should be exercised in interpreting our data. First, although our sample size was relatively large, the time course of our study was relatively long and different levels of emergency may exist. Furthermore, the low rate of mortality among our patients may have exaggerated the predictive power of our models. A second limitation of our study is that although we demonstrated the potential prognostic power of coagulation tests used in combination with parameters obtained at admission, the technology and methodology we used to assess coagulation tests cannot be readily obtained at admission.

We believe our findings highlight the importance of including coagulation test results in state-of-the-art outcome prediction models and set the stage for using this approach in future large-scale clinical trials. Moreover, we believe our results pave the way for the development of tools that connect basic science and clinical research with clinical evidence-based decision making that will ultimately improve the care of patients with TBI.

## Conclusion

Coagulopathy is commonly observed after TBI and is associated with the severity of brain injury. Coagulation tests can improve the predictive power of the standard model for in-hospital mortality after TBI.

## References

[1] Hyder AA, Wunderlich CA, Puvanachandra P, Gururaj G, Kobusingye OC (2007). The impact of traumatic brain injuries: a global perspective. NeuroRehabilitation.

[2] Dewall J (2010). The ABCs of TBI. Evidence-based guidelines for adult traumatic brain injury care. JEMS.

[3] de Oliveira Manoel AL, Neto AC, Veigas PV, Rizoli S (2015). Traumatic brain injury associated coagulopathy. Neurocrit Care.

[4] Wafaisade A, Lefering R, Tjardes T (2010). Acute coagulopathy in isolated blunt traumatic brain injury. Neurocrit Care.

[5] Laroche M, Kutcher ME, Huang MC, Cohen MJ, Manley GT (2012). Coagulopathy after traumatic brain injury. Neurosurgery.

[6] Cap AP, Spinella PC (2011). Severity of head injury is associated with increased risk of coagulopathy in combat casualties. J Trauma.

[7] Lustenberger T, Talving P, Kobayashi L (2010). Time course of coagulopathy in isolated severe traumatic brain injury. Injury.

[8] Genet GF, Johansson PI, Meyer MA (2013). Trauma-induced coagulopathy: standard coagulation tests, biomarkers of coagulopathy, and endothelial damage in patients with traumatic brain injury. J Neurotrauma.

[9] Harhangi BS, Kompanje EJ, Leebeek FW, Maas AI (2008). Coagulation disorders after traumatic brain injury. Acta Neurochir.

[10] Epstein DS, Mitra B, Cameron PA, Fitzgerald M, Rosenfeld JV. Acute traumatic coagulopathy in the setting of isolated traumatic brain injury: definition, incidence and outcomes. Br J Neurosurg. 2014:1–5. doi:10.3109/02688697.2014.950632. 10.3109/02688697.2014.950632 25153987

[11] Epstein DS, Mitra B, O'Reilly G, Rosenfeld JV, Cameron PA (2014). Acute traumatic coagulopathy in the setting of isolated traumatic brain injury: a systematic review and meta-analysis. Injury.

[12] Van Beek JG, Mushkudiani NA, Steyerberg EW (2007). Prognostic value of admission laboratory parameters in traumatic brain injury: results from the IMPACT study. J Neurotrauma.

[13] Kushimoto S, Yamamoto Y, Shibata Y, Sato H, Koido Y (2001). Implications of excessive fibrinolysis and alpha(2)-plasmin inhibitor deficiency in patients with severe head injury. Neurosurgery.

[14] West KL, Adamson C, Hoffman M (2011). Prophylactic correction of the international normalized ratio in neurosurgery: a brief review of a brief literature. J Neurosurg.

[15] Yuan F, Ding J, Chen H (2012). Predicting outcomes after traumatic brain injury: the development and validation of prognostic models based on admission characteristics. The journal of trauma and acute care surgery.

[16] Kuo JR, Chou TJ, Chio CC (2004). Coagulopathy as a parameter to predict the outcome in head injury patients--analysis of 61 cases. Journal of clinical neuroscience: official journal of the Neurosurgical Society of Australasia.

[17] Steyerberg EW, citations LHFP (2008). Validating prediction models. BMJ.

[18] Perel P, Edwards P, Wentz R, Roberts I (2006). Systematic review of prognostic models in traumatic brain injury. BMC medical informatics and decision making.

[19] Mushkudiani NA, Hukkelhoven CW, Hernandez AV (2008). A systematic review finds methodological improvements necessary for prognostic models in determining traumatic brain injury outcomes. J Clin Epidemiol.

[20] Maas AI, Lingsma HF, Roozenbeek B (2015). Predicting outcome after traumatic brain injury. Handb Clin Neurol.

[21] Collaborators MCT, Perel P, Arango M (2008). Predicting outcome after traumatic brain injury: practical prognostic models based on large cohort of international patients. BMJ.

[22] MacLeod JB, Lynn M, McKenney MG, Cohn SM, Murtha M (2003). Early coagulopathy predicts mortality in trauma. J Trauma.

[23] Halpern CH, Reilly PM, Turtz AR, Stein SC (2008). Traumatic coagulopathy: the effect of brain injury. J Neurotrauma.

[24] McCully SP, Schreiber MA (2013). Traumatic brain injury and its effect on coagulopathy. Semin Thromb Hemost.

[25] Allard CB, Scarpelini S, Rhind SG (2009). Abnormal coagulation tests are associated with progression of traumatic intracranial hemorrhage. J Trauma.

[26] Talving P, Benfield R, Hadjizacharia P (2009). Coagulopathy in severe traumatic brain injury: a prospective study. J Trauma.

[27] Schnuriger B, Inaba K, Abdelsayed GA (2010). The impact of platelets on the progression of traumatic intracranial hemorrhage. J Trauma.

[28] Engstrom M, Romner B, Schalen W, Reinstrup P (2005). Thrombocytopenia predicts progressive hemorrhage after head trauma. J Neurotrauma.

[29] Zehtabchi S, Liu T (2008). Effect of hypoperfusion and the protein C pathway on the early coagulopathy after traumatic brain injury. J Trauma.

